# Influence of Age and Dose on the Effect of Resveratrol for Glycemic Control in Type 2 Diabetes Mellitus: Systematic Review and Meta-Analysis

**DOI:** 10.3390/molecules27165232

**Published:** 2022-08-16

**Authors:** Beatriz Isabel García-Martínez, Mirna Ruiz-Ramos, José Pedraza-Chaverri, Edelmiro Santiago-Osorio, Víctor Manuel Mendoza-Núñez

**Affiliations:** 1 Research Unit on Gerontology, FES Zaragoza, National Autonomous University of Mexico, Mexico City 09230, Mexico; 2 Department of Biology, Faculty of Chemistry, National Autonomous University of Mexico (UNAM), Mexico City 04510, Mexico; 3 Hematopoiesis and Leukemia Laboratory, Research Unit on Cell Differentiation and Cancer, FES Zaragoza, National Autonomous University of Mexico, Mexico City 09230, Mexico

**Keywords:** resveratrol, glucose, glycated hemoglobin, insulin resistance, dose, age

## Abstract

Background: Several clinical trials have suggested that resveratrol has hypoglycemic properties; however, there are other studies in which such an effect has not been observed. Methods: We carried out a systematic search in several databases; seventeen studies were selected for the systematic review and fifteen were included in the meta-analysis. Results: Resveratrol decreases glucose levels in subjects aged 45–59 years at doses <250 mg/day (−8.64 mg/dL, *p* < 0.00001), 250–500 mg/day (−22.24 mg/dL, *p* = 0.0003), and 500–1000 mg/day (−28.40 mg/dL, *p* = 0.0008), while in subjects older than 60 years, it only decreases with doses of 250–500 mg/day. Likewise, HbA1c improved in subjects aged 45–59 years with doses of 250–500 mg (−0.60%, *p* < 0.00001), but not in subjects older than 60 years. Insulin levels improved in subjects aged 45–59 years with doses < 250 mg/day (−0.80 mIU/L, *p* = 0.0003) and doses of 250–500 mg/day (−5.0 mIU/L, *p* = 0.0003), although in subjects older than 60 years, they only improved with doses of 250–500 mg/day (−1.79 mIU/L, *p* = 0.01). On the other hand, HOMA-IR only improved in subjects older than 60 years with doses of 250–500 mg/day (−0.40, *p* = 0.01). Conclusions: Resveratrol has a statistically significant dose–response effect on glucose concentrations, HbA1c, and insulin levels; however, there is not enough scientific evidence to propose a therapeutic dose.

## 1. Introduction

Type 2 diabetes mellitus (T2DM) represents a serious public health problem worldwide owing to its high prevalence among the adult population. T2DM triggers the appearance of micro and macrovascular complications, making it necessary to implement complementary therapeutic strategies to decrease blood glucose levels [1,2]. In this sense, the therapeutic usefulness of naturally occurring compounds with hypoglycemic properties has been investigated, among which resveratrol (RV) stands out [3]. Antioxidant, anti-inflammatory, and hypoglycemic effects are attributed to RV, because it has been observed that, in cell cultures and in animal models, it improves insulin sensitivity and reduces blood glucose [4,5]. It has been proposed that RV could improve pancreatic β-cell functionality by protecting them from oxidative damage and decreasing the production of pro-inflammatory cytokines in the islets of Langerhans, restoring β-cell secretory functions and normalizing insulin secretion [6,7]. In this sense, several clinical trials have suggested that RV increases insulin sensitivity and decreases blood glucose levels in subjects with T2DM, and the same effect has been observed in subjects with insulin resistance [8,9]. However, there are other clinical trials in which the therapeutic effects of RV have not been observed. These inconsistencies have been attributed to the low bioavailability of the compound and to the wide range of doses used, as doses from 5 mg to 5000 mg/day have been used [8]. Likewise, age relative to aging is a factor that influences effectiveness, as biological reserve and efficiency decrease with aging, so inflammation and oxidative stress increase significantly as age increases from the fifth decade of life [10,11]. 

In accordance with the above, in a systematic review and meta-analysis previously published by our research team, we found that, after oral administration of RV, glucose, insulin, HbA1c, and HOMA-IR levels decrease in subjects with T2DM; however, the age-related effective dose could not be ascertained [12]. In this regard, it has been proposed that the dose of RV plays a crucial role, because, according to the biological mechanism of hormesis, an antioxidant or pro-oxidant effect could be present depending on the dose administered [13,14]. This type of inverse response to varied doses administered to the same individual has been observed with various drugs, allowing the therapeutic doses to be specified based on the desired effect, which can be beneficial or harmful because both are possible depending on the situation [15,16]. This biphasic response rules out dose linearity and response threshold models, helping to establish the therapeutic use of different drugs [17,18]. Given this, it has been suggested that RV could trigger opposite responses depending on the dose used, a phenomenon that has been observed with other nutraceuticals, such as vitamins C and E, which, at low doses, act as antioxidants, but at high doses, act as pro-oxidants [19]. Regarding age, our previously published systematic review found that age could also influence the therapeutic effects of RV. In this regard, it was found that, in individuals under 60 years old, RV significantly decreases glucose, insulin, and HbA1c levels, which was not observed in subjects over 60 years old [12]. In this context, its well-known that physiological function declines with aging, which arises as a result of the interaction of several cellular and molecular mechanisms, such as oxidative stress, inflammation, and cellular senescence, among others, whose processes interact additively and even synergistically, and thereby alter the normal functioning of cells, with subsequent damage to tissues, organs, and systems [20,21]. 

An aging-related alteration occurs in the gastrointestinal system, where the number of functional intestinal epithelial cells decreases significantly as a result of molecular alterations that induce cell senescence or apoptosis, significantly affecting the gastrointestinal absorption of substances, including drugs and nutraceuticals such as RV. This would directly impact the effects it exerts, so higher doses than those indicated for young people would be required to reach the blood concentration with a therapeutic effect in older adults [21,22]. On the other hand, higher doses of RV are also required in older subjects because of the large number of altered pathways that occur during the aging process, which could include one or more RV target molecules, causing a large portion of RV to be consumed and leaving a small amount to exert hypoglycemic effects [21].

Considering the above, the purpose of this systematic review and meta-analysis is to present a synthesis of knowledge on the differentiated effect of RV considering dose and age on the glycemic control of T2DM.

## 2. Results

### 2.1. Literature Search

**Figure 1:**
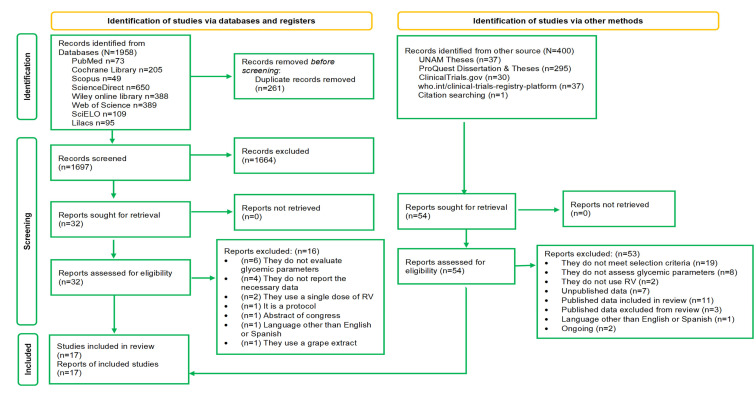
Study selection flow chart.

A total of 1958 records were identified from databases and 400 from other sources (Figure 1). Duplicate records (261 records identified from databases and 15 records from other sources) were eliminated, and after a review of titles and abstracts, those that did not meet the selection criteria were discarded. The number of eligible records from the databases was 32, while 54 potentially includable records were selected from other sources. Documents were retrieved from the 86 preselected records, as shown in Figure 1. After a detailed review of the full text, 16 documents were excluded from databases (Appendix A) and 53 from other sources (Appendix B), leaving only 17 studies that were included in the systematic review; however, two studies were excluded from the meta-analysis owing to insufficient data.

### 2.2. Study Characteristics

**Figure 2:**
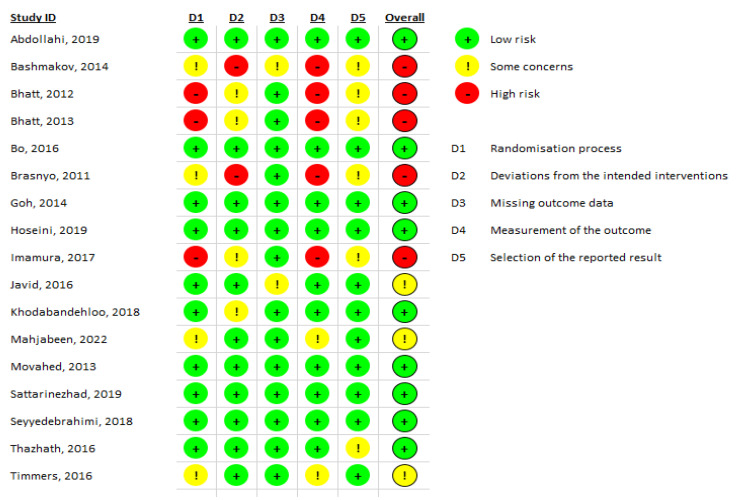
Assessment of the risk of bias (Robins-2 Beta version, Cochrane) [23,24,25,26,27,28,29,30,31,32,33,34,35,36,37,38,39].

Among the included studies, 12 had a double-blind parallel design, 2 were double-blind crossover, 1 had a parallel-blind design, and 2 were open-label. The number of individuals with T2DM included in the systematic review and meta-analysis was 921, aged between 50 and 68 years. The minimum number of subjects included in each study was 10 and the maximum was 179; the minimum dose of RV was 10 mg/day and the maximum was 3 g/day. The duration of the interventions ranged from 4 weeks to 6 months. Table 1 summarizes the characteristics of the studies included in the systematic review and certainty assessment (GRADE). Figure 2 presents the general result of the risk of bias assessment.

### 2.3. Meta-Analysis

Fifteen effect sizes on glucose concentrations, 12 effect sizes on HbA1c, 11 effect sizes on insulin levels, and 11 effect sizes on HOMA-IR were included in the meta-analysis. To evaluate the influence of the age of the participants and the dose of RV used on its therapeutic effect, analyses stratified by dose of RV and age of the participants were carried out (Table 2). In addition, a combined analysis by age and dose was performed, owing to the heterogeneity found in the included studies. In the analysis by dose, the included studies were categorized into four groups: (i) doses < 250 mg/day, (ii) doses of 250–500 mg/day; (iii) doses > 500–1000 mg/day; and (iv) doses > 1000 mg/day. The results suggest that glucose concentrations and HbA1c percentage decrease significantly if doses of 250–500 mg/day are administered (−20.72 mg/dL, *p* < 0.0001; −0.42%, *p* < 0.001). Meanwhile, insulin levels improve with RV doses < 250 mg/day (−1.22 mIU/L, *p* < 0.0001). HOMA-IR remained unchanged. Additionally, dose–response regression analyses were performed to assess the effect of the daily dose of RV on serum levels of glucose, insulin, HbA1c, and HOMA-IR (Appendix C), in which we observed a statistically significant relationship between the dose of RV with HbA1c (R^2^ = 0.22, *p* < 0.05). 

The analysis carried out according to the age of the participants reveals that glucose concentrations, percentage of HbA1c, and insulin levels improve significantly in subjects between 45 and 59 years (*p* < 0.01); insulin levels also improve significantly in individuals 60 years and over, while HOMA-IR decreases significantly in individuals aged 60 years and over (*p* = 0.007), despite the fact that no changes are observed globally.

Finally, in the combined analysis by age and dose, we observed that, from doses lower than 250 mg and up to 1000 mg of RV, glucose levels decrease in subjects between 45 and 59 years and the effect is more evident at doses of 500–1000 mg (<250 mg: −8.64 mg/dL, *p* < 0.0001, 250–500 mg: −22.24 mg/dL, *p* = 0.0003, 500–1000 mg: −28.40, *p* = 0.0008). We also found that HbA1c decreased significantly using doses of 250–500 mg in individuals aged 45–59 years (−0.60%, *p* < 0.00001); and insulin levels decrease by administering doses < 250 mg and between 250 and 500 mg of RV in subjects aged 45–59 years (−0.80 mIU/L, *p* = 0.0003; −5.0 mIU/L, *p* < 0.0003), while in people 60 years and over, it decreases only with doses < 250 mg/day. Regarding HOMA-IR, even though the global result does not show RV effects, in the combined analysis by age and dose, we observed that insulin resistance decreases in subjects 60 years and over (−0.40, *p* = 0.01) using a 250–500 mg RV dose. Egger’s test did not show the presence of publication bias.

## 3. Discussion

T2DM is a health problem that requires immediate attention given its high prevalence in the population, which highlights the need to design strategies that allow delaying the appearance of micro and macrovascular complications that accompany it [40,41]. For this reason, nutraceutical compounds with antioxidant and anti-inflammatory properties have been used to attenuate OS and the inflammation that occurs in people with T2DM and slow down the development of cardiovascular diseases [42,43,44]. Among the nutraceutical compounds, RV stands out, a polyphenol that has been extensively studied to evaluate its antioxidant and anti-inflammatory capacity, as well as hypoglycemic capacity [45]. In the last decade, several clinical trials have been carried out to evaluate the therapeutic efficacy of RV in glycemic control; however, the results are controversial [46,47].

In the present systematic review and meta-analysis, the effect of RV supplementation on markers of glycemic control in subjects with T2DM was evaluated. We found that glucose levels decrease considerably after oral administration of RV, in agreement with the meta-analyses of Zhang et al. (2021) [48] and Liu et al. (2014) [49], as well as with the results previously found by our research group [12]. Our results suggest that RV significantly improves the percentage of HbA1c, which coincides with Hausenblas et al. (2015) [50], who, after performing a meta-analysis, found that RV exerts a beneficial effect on the percentage of HbA1c in diabetic subjects. Similarly, we observed that the administration of RV has a beneficial impact on insulin levels, although it does not modify insulin resistance—partially similar results to those observed by Liu et al. (2014) [49], whose meta-analysis showed that RV consumption significantly reduces glucose and insulin concentrations, in addition to decreasing the HbA1c percentage and improving insulin resistance in diabetic subjects.

Despite the above, our conclusions are opposed to the results of the meta-analysis carried out by Jeyaraman et al. (2020) [51]. They found that RV does not exert beneficial effects on glucose, HbA1c, and insulin levels. These conflicting results are probably due to the difference between the number of studies included by Jeyaraman et al. (2020) [51] and our research team (3 vs. 15), a situation that results from the disparity in the employee selection criteria, with ours being broader, as occurs in the meta-analyses carried out by Hausenblas et al. (2015) [50] and Liu et al. (2014) [49].

**Figure 3:**
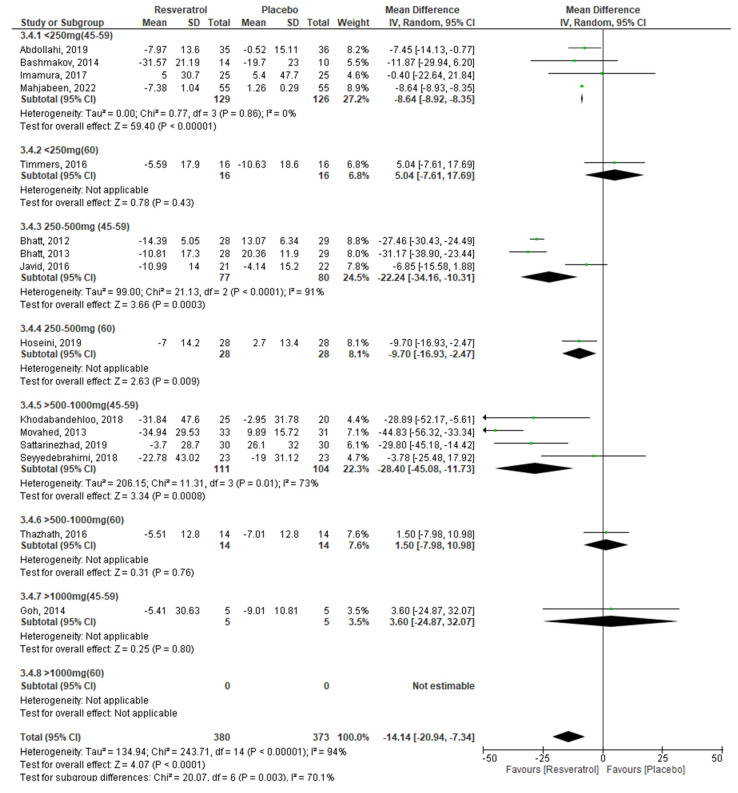
Effect of RV on glucose, stratified by age and dose.

**Figure 4:**
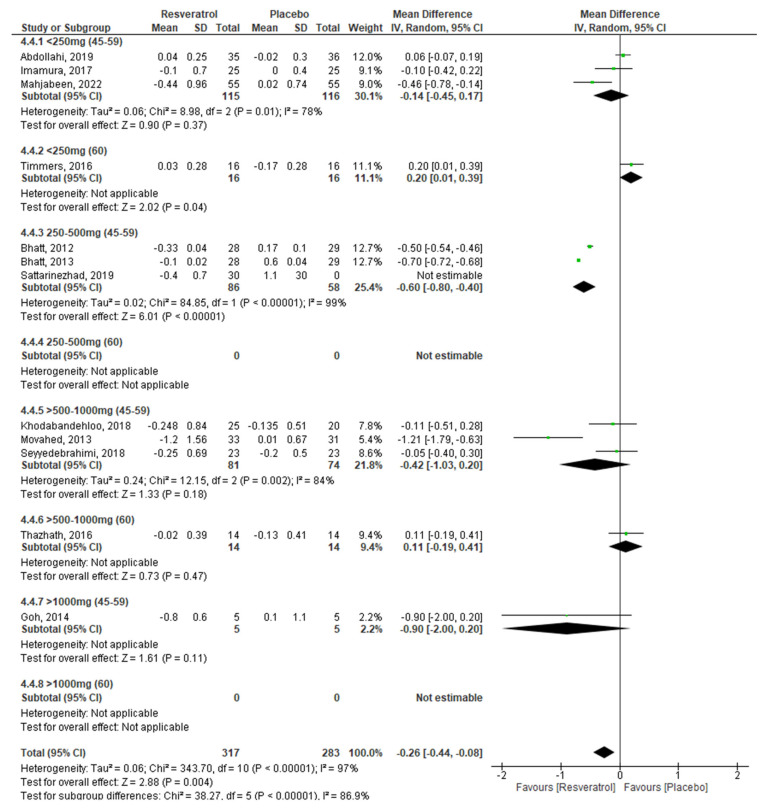
Effect of RV on HbA1c, stratified by age and dose.

**Figure 5:**
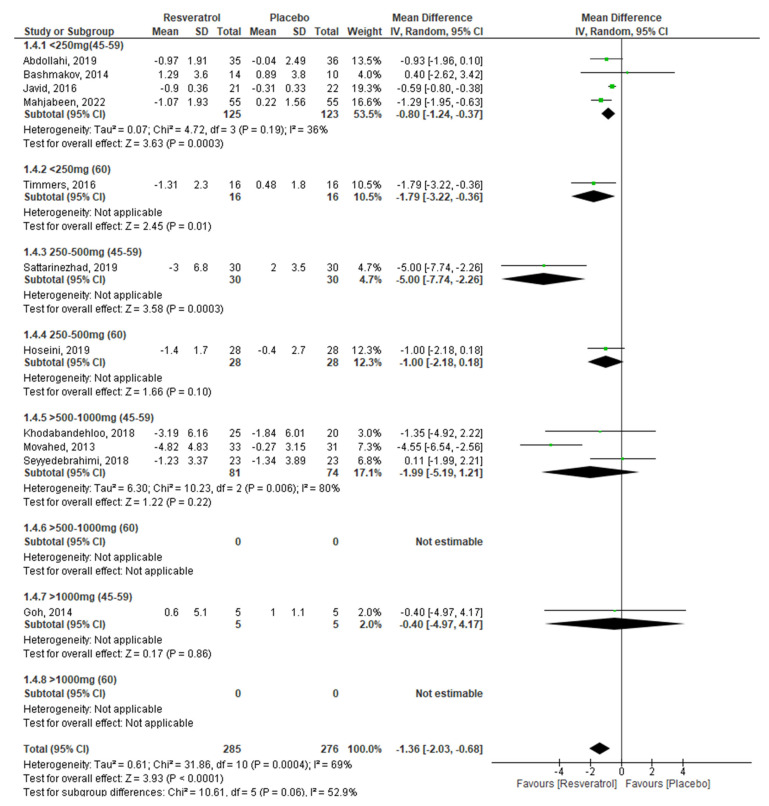
Effect of RV on insulin, stratified by age and dose.

**Figure 6:**
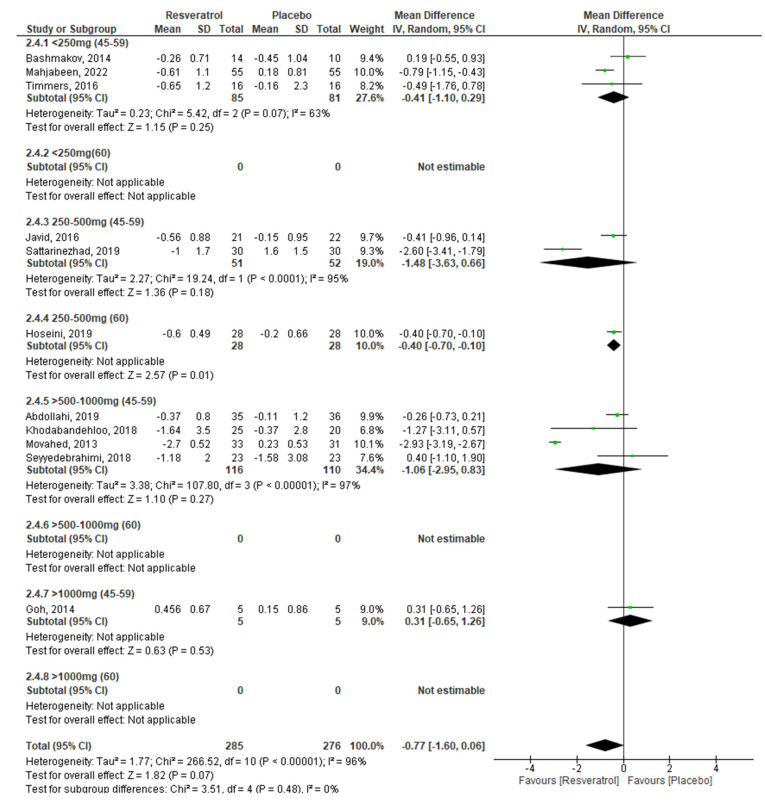
Effect of RV on HOMA-IR, stratified by age and dose.

To meet the proposed objectives, it was necessary for the analyses of the different glycemic markers to be stratified by dose and age of the participants (Table 2), in addition to performing a combined analysis by age and dose of RV, as shown in Figure 3, Figure 4, Figure 5 and Figure 6.

### 3.1. Effect of RV on Glycemic Control by Dose

The analysis by dose, presented in Table 2, shows a significantly beneficial effect of RV consumption on glucose concentrations, insulin levels, and the percentage of HbA1c at doses of 250–500 mg/day; insulin also improves at doses < 250 mg/day. In this sense, it has been documented that the efficacy of RV can be modified, as the molecular target changes depending on the dose [51]. In addition to this, it is considered that RV exhibits the effect of hormesis, that is, its therapeutic effects are presented as a dose–response relationship. This implies that low doses of RV stimulate a response, while high doses inhibit it [52]. In this regard, it has been shown that the histone deacetylase called sirtuin 1 (SIRT1), one of the main molecular targets of the RV, modulates several signaling pathways that regulate metabolic activities. One of them is the activation of AMP-dependent kinase (AMPK), which is directly involved in energy metabolism because it stimulates mitochondrial biogenesis and function, thus inducing glucose uptake and subsequent use [53]. Some studies have shown that moderate doses of RV activate SIRT1 and AMPK, while high doses activate AMPK independently of SIRT1 without affecting mitochondrial function, which translates into less glucose uptake by cells and thus an increase in blood [54,55].

The hormetic effect of RV is due to the fact that the agonist molecules activate two or more subtypes of receptors with different binding affinity, and then the receptor–agonist union would give rise to different responses with different magnitude, in such a way that some receptor subtypes would induce certain pathways, while others would inhibit them. It has also been proposed that agonists can bind to a single type of receptor, but at different sites, triggering very different responses [56,57]. For this reason, the dose of RV can cause different responses depending on the availability of the agonist, so if there is a low concentration of RV, it could bind to a single receptor, but if there is a high amount of the compound, the binding could occur in two or more subtypes, giving rise to different responses [57,58]. In addition, it is necessary to consider the time factor, as it can be of vital importance in the hormetic response, because, depending on the time of exposure to a substance, an immediate response or a delayed response that arises as a result of an adaptive process can be generated. However, studying the phenomenon of hormesis considering the dose–response–time relationship is complicated, as it would be necessary to use a wide variety of RV doses and monitor them at different times, which, in addition to being complicated, would be very expensive [56,57,58].

Regarding hypoglycemic effects, we have mentioned that RV activates AMPK through the activation of SIRT1, increasing glucose uptake. In addition, SIRT1 is capable of deacetylating and inactivating the Forkhead box-O1 protein (FOXO1), thereby inhibiting pancreatic β-cell apoptosis and triggering an increase in insulin production, the final result of which is an increase in glucose uptake [55,59]. RV also increases the synthesis and expression of insulin-dependent glucose transporter (GLUT4), increasing glucose internalization by cells [59]. Another molecular target of RV is the receptor for advanced glycation end products (RAGE), which is capable of phosphorylating the serine/threonine fragment of the insulin receptor (IR), altering its protein structure and inhibiting its binding to insulin. The expression of RAGE is decreased by the effect of RV [59]. The conjunction of these mechanisms induced by RV produces several results: it (i) increases the synthesis of insulin; (ii) increases the internalization, use, and storage of glucose; (iii) improves sensitivity to insulin; and (iv) restores insulin binding to IR, managing to reduce glycemia and gradually reduce HbA1c levels. Considering the above, our findings are partially similar to those observed by Zhu et al. (2017) [60], who found that doses < 100 mg/day do not modify glucose concentrations, while higher doses, even 1000 mg/day, significantly decrease glucose concentrations in individuals with T2DM.

On the other hand, RV administered at relatively low doses has also been shown to improve insulin sensitivity, in addition to decreasing insulin secretion when administered over the long term [61]. Similar effects have been observed after short-term, high-dose RV administration [62]. The present meta-analysis denoted that insulin levels decrease after the administration of doses between 250 and 500 mg/day of RV, but also do so at low doses of RV (<250 mg/day), which coincides with what was previously mentioned. 

Regarding HOMA-IR, we did not observe significant changes related to the dose used, which is consistent with the findings of Zhu et al. (2017) [60] and Jeyaraman et al. (2020) [63].

### 3.2. Effect of RV on Glycemic Control by Age

In the analysis by age (Table 2), it was observed that glucose concentrations, HbA1c percentage, and insulin levels improve significantly in subjects aged 45–59 years, while HOMA-IR shows significant changes in subjects aged 60 years and over. Such results are partially similar to what was observed by Crandall et al. (2012) [62], whose meta-analysis found that oral administration of RV significantly improves insulin sensitivity and decreases glucose levels in older adults. In this context, among the studies included in this review that were carried out in adults over 60 years old, only the one carried out by Hoseini et al. (2019) [38] found a statistically significant decrease in serum glucose after RV administration. Meanwhile, of the studies carried out on people between 45 and 59 years of age, the studies by Brasnýo et al. (2011) [23], Goh et al. (2014) [34], Imamura et al. (2017) [25], and Seyyedebrahimi et al. (2018) [31] did not report significant changes in the glycemic parameters evaluated; however, it is necessary to mention that Brasnýo et al. (2011) [23] and Imamura et al. (2017) [25] used very low doses of RV, while Goh et al. (2014) [34] used a very high dose and, in all three studies, the duration was relatively short (<3 months), which explains the positive effect of low doses for long periods, in contrast to the effect of doses higher than 2.5 g, which no longer exhibit beneficial effects, and may even be harmful [61]. Regarding the study by Seyyedebrahimi et al. (2018) [31], despite using a dose of 800 mg/day, the duration of the intervention is short (8 weeks) and the sample size is relatively small, so these two factors are considered to be largely responsible for the lack of RV effect.

As the analysis by age does not provide sufficient evidence to propose an effective dose, a combined analysis by age and dose was performed, as described below.

### 3.3. Effect of RV on Glycemic Control by Age and Dose

This combined analysis by age and dose showed that serum glucose levels improve in subjects aged 45–59 years using a maximum dose of 1000 mg/day, as higher doses do not exhibit any effect on glucose (Figure 3). This agrees with the results of Zhang et al. (2021) [48], Liu et al. (2014) [49], Zhu et al. (2017) [60], and Guo et al. (2018) [64], who, in their respective meta-analyses, observed a significant decrease in blood glucose concentrations. Furthermore, these studies were mostly conducted in people under 60 years old. Meanwhile, in subjects aged 60 years and over, beneficial effects of RV on blood glucose were observed at doses of 250–500 mg/day, although only one study was included in this subgroup. In addition, it was not possible to estimate the effect at doses greater than 1000 mg/day in this age group because of the scarcity of studies carried out. Our results are similar to what was observed by Jeyaraman et al. (2020) [63], whose meta-analysis shows no beneficial effects of RV on blood glucose in subjects 60 years and over.

Regarding the percentage of HbA1c (Figure 4), we observed a statistically significant decrease in subjects aged 45–59 years who were administered doses between 250 and 500 mg/day, but no beneficial effects found using other doses, which coincides with the results of the meta-analyses by Husenblas et al. (2015) [50], Zhu et al. (2017) [60], and Guo et al. (2018) [64], where an improvement in HbA1c is observed. However, in subjects aged 60 years and over, no beneficial effects of RV administration on HbA1c were observed, results that coincide with those found in the meta-analysis by Jeyaraman et al. (2020) [63].

Regarding insulin levels, beneficial effects of RV were observed using doses of 250–500 mg/day in subjects aged 45–59 years, as occurred with the percentage of HbA1c, and there were also significant changes at doses < 250 mg/day. These results coincide with what was observed in the meta-analyses carried out by Zhang et al. (2021) [48] and Zhu et al. (2017) [60], whose results show a considerable improvement in insulin levels after oral administration of resveratrol. We found significant changes in insulin levels in subjects 60 years and over at doses < 250 mg/day, although only one study was included in this dose subgroup. Our results are partially consistent with what was observed by Hausenblas et al. (2015) [50].

Finally, the analysis to observe the effects of RV on insulin resistance (HOMA-IR) showed that this marker improves considerably in individuals aged 60 and over who received doses of RV ranging from 250 to 500 mg/day. These results agree with those of Zhang et al. (2021) [48], Liu et al. (2014) [49], and Zhu et al. (2017) [60].

As can be seen in the results, favorable effects of oral administration of RV are observed in individuals aged 60 years and over on insulin levels and HOMA-IR, although these effects are very small compared with those observed in younger individuals. Therefore, they lack clinical relevance. In this context, it is known that one of the main molecular targets of RV is SIRT1, a protein that plays a key role in glucose metabolism, and whose activity decreases as the aging process progresses, which is why the response of organisms aged before RV may be diminished [65,66], which would explain the lack of effect in adults older than 60 years. Because of this, the use of higher doses of RV has been proposed, because, together with its low bioavailability, we find a diminished response on the part of older adults, who largely present absorption problems at the intestinal level, as well as a lower ability to metabolize RV, which suggests the need to use higher doses than those that are effective in younger individuals [67]. In this sense, during aging, organisms undergo a series of changes at the molecular and cellular level, which lead to a decrease in the ability to maintain homeostasis, which is reflected through a decrease in physiological functions, including the metabolism of substances such as RV [10]. Among the molecular and cellular changes that could affect the response of aging organisms to RV, we find cellular senescence, mitochondrial dysfunction, alterations in proteostasis, as well as oxidative stress (OS) and inflammation [68].

Cellular senescence is a state of suppressed proliferation, in which metabolic functions and cell viability are maintained [69]. This is because of the fact that telomeres wear out and shorten their length in each cell cycle, so that, after a certain number of cell divisions, a response mechanism is activated in the face of possible DNA damage, the main consequence of which is the arrest of the cycle cell to limit the spread of damage [70]. Senescence may also be due to damage caused by stressors such as OS that occurs in aging subjects [71]. Regardless of the cause, cells lose their proliferative capacity and exhibit some metabolic and morphological changes, which give them the ability to alter the tissues of which they are a part and decrease or modify their functionality. Such is the case of the tissues involved in the absorption of substances such as RV, which would explain, in part, the lower uptake of the compound in subjects aged 60 years and over and, therefore, the decreased response to it [72]. Regarding mitochondrial dysfunction, it is well known that the mitochondria are responsible for energy production of cells, a function that is altered during aging [73]. This occurs as a result of the excessive production of reactive oxygen species (ROS), which damage mitochondrial components and reduce their ability to supply energy [74]. Although defective mitochondria are eliminated through a process known as mitophagy, aged cells have a large number of damaged mitochondria because of the inefficiency of mitophagy, the result of which is the release of mitochondrial DNA into the cytoplasm and the subsequent activation of senescence cellular [75], which derives in alterations in the functionality of the tissues. 

In this sense, it is known that RV, through the activation of SIRT1, is able to restore mitophagy and induce mitochondrial biogenesis, thereby increasing glucose uptake and energy production; however, it is possible that the administered dose of RV is not sufficient to induce mitochondrial biogenesis, as the compound would be mainly responsible for counteracting the damage caused by ROS, which, together with the cellular senescence that takes place in individuals of 60 years and over, would require a higher dose [76,77]. Regarding the alterations of proteostasis, it has been documented that, because proteins modulate various metabolic pathways, as well as the alterations they suffer such as misfolding, oxidation, and aggregation, and the reduced capacity of cells to eliminate damaged proteins, they are essential to understand the deterioration in the functionality of various tissues and organs, an event that takes place in aged individuals [78]. The aging of organisms is characterized by the presence of a considerable number of damaged or oxidized proteins, which are produced as a consequence of alterations in protein homeostasis, also called proteostasis, which can be defined as the balance of structural and biochemically functional proteins [79]. The accumulation and aggregation of damaged proteins increases in parallel with age, which directs human cells towards a senescence process, with the respective alteration of the functionality of the tissues [80]. In addition, protein misfolding can alter various metabolic pathways, including those induced by SIRT1 and that play a crucial role in glucose uptake, partially explaining the lack of effect of RV in subjects aged 60 years and over, as observed in this meta-analysis.

As has been pointed out, during aging, there is an excessive production of ROS, coupled with a decrease in the activity of enzymes and antioxidant mechanisms of aged organisms, triggering OS [81]. This condition causes damage to different macromolecules such as lipids, proteins, and DNA, as well as mitochondrial dysfunction [82]. In addition, ROS are able to induce the synthesis of proinflammatory cytokines through the activation of the nuclear transcription factor κB (NF-κB), which gradually results in the development of low-grade chronic inflammation, called inflammaging, whose presence favors damage at the molecular, organ, and systemic levels [83]. It has been documented that there is a close interconnection between OS and inflammation, as the existence of one of them induces the other, establishing a vicious circle that leads to cell damage, and this in turn causes the cell to enter into a state of senescence to avoid the proliferation of the damage caused [84]. As a result, the functionality of the cells is altered, as well as that of the tissues of which they are a part, which is why it is probable that they do not take up RV in sufficient quantity to induce a favorable response.

It is important to highlight the hormetic effect of RV, as the dual response of a cell to different doses of a compound can be interpreted as an adaptive mechanism of the same cell to compensate for any imbalance in homeostasis, as occurs in aging, thus understanding the mechanisms involved in hormesis would help to precisely define the appropriate doses and target molecules that allow the design of effective strategies to assist in the treatment of diseases associated with old age, such as T2DM and its complications [14].

Among the most relevant limitations of this study, we can point out that five of the included studies have a high risk of bias, which could affect the interpretation of the results. In addition, in the evaluation of certainty (GRADE), we observed that 10 studies provided a low or very low certainty of evidence, which directly impacts the results obtained, so it is necessary to interpret them carefully. Finally, the small number of studies carried out in people aged 60 years and over did not allow analysis with doses higher than 1000 mg/day and, in some cases, such as HOMA-IR and insulin, it was not possible to analyze the effect of RV with doses between 500 and 1000 mg/day, given the scarcity of studies in subjects belonging to this age group. In this way, the evidence provided by our meta-analysis is not sufficient to propose a possible effective dose of RV based on age, although it allows us a glimpse that, in subjects over 60 years of age, the dose used plays a crucial role in the effect of the compound.

## 4. Materials and Methods

This systematic review and meta-analysis was carried out following the guidelines for the presentation of systematic reviews and meta-analyses (PRISMA 2020) [85]. The protocol was previously registered in PROSPERO (CRD42021227865).

### 4.1. Search Strategy

Two authors (G.-M. B.I. and P.-C. J.) searched the following databases: PubMed-Medline, Scopus, Cochrane library, Web of Science, Wiley online library, ScienceDirect, SciELO, and LILACS; the search was carried out until 31 January 2022, and was performed again prior to the final analysis of the results. The search strategy for the Cochrane Library, Web of Science, and Scopus was as follows: “Resveratrol AND (glycemic control OR fasting blood glucose OR insulin resistance OR insulin levels, OR glycated hemoglobin OR diabetes mellitus OR diabetics)”. For the search in PubMed, the same strategy was used and the filter “clinical trials” was additionally activated. In ScienceDirect, the same strategy was used again, but the filter “research articles” was activated, and it was indicated that the titles always included the term “resveratrol”. The Wiley online library search was performed using the aforementioned strategy and the “journals” filter was additionally activated. For the search in SciELO and LILACS, only the term “resveratrol” was used. Additionally, a search of UNAM theses, ProQuest Dissertation and Theses, ClinicalTrials.gov, and who.int/clinical-trials-registry-platform was performed to identify unpublished studies, but potentially included in the systematic review and meta-analysis. For the UNAM theses, the keyword “Resveratrol” was used; in ProQuest Dissertations and Theses, “Resveratrol AND type 2 diabetes mellitus” was used, specifying that the following keywords be included in the title: diabetes, metabolism, insulin resistance, type 2 diabetes, resveratrol, insulin, glucose, or diabetes mellitus. In ClinicalTrials.gov, “Diabetes” was placed in condition or disease and “Resveratrol” in other terms, while for the search in who.int/clinical-trials-registry-platform, the terms “Resveratrol AND diabetes” were used. Subsequently, two reviewers (S-O. E. and R-R. M.), independently evaluated the identified titles and abstracts, and discrepancies were resolved by a third reviewer (M-N. V.M.). After selecting the titles and abstracts that met the established criteria, the full texts of the chosen articles were retrieved and carefully reviewed to make a final selection of the studies included in the review.

### 4.2. Selection of Studies

To select the publications of the systematic review and meta-analysis, the following criteria were used:

#### 4.2.1. Selection Criteria

- Randomized clinical trials (RCTs) blind, double blind, open, or crossover
- Oral administration of RV as a coadyuvant in T2DM treatment
- Studies with placebo group as comparator
- Published in English and/or Spanish
- Evaluate at least one of the following glycemic markers: serum glucose and insulin levels, HbA1c, and insulin resistance (HOMA-IR)
- Intervention lasting at least 2 weeks
- Carried out in individuals over 20 years of age with T2DM

#### 4.2.2. Exclusion Criteria

- Use of RV in combination with other compounds
- Use of compounds derived from resveratrol
- Investigations without a comparator
- Conference summaries

### 4.3. Assessment of the Risk of Bias and Certainty of the Included Studies

After reviewing the full texts, those that did not meet the established criteria were eliminated. Likewise, the risk of bias was assessed using the risk of bias assessment tool Robins-2 beta version of the Cochrane collaboration [86]. The following items were considered: randomization process, deviations from the intended interventions, missing outcome data, measurement of the outcome, and selection of the reported result. The certainty of the evidence was evaluated using the GRADE approach, which considers the existence of risk of bias, inconsistency in results, uncertainty about whether the evidence is direct, imprecision, and publication bias as factors that decrease the certainty of the evidence, while factors such as a strong association, the presence of a dose–response gradient, and evidence that confounding factors could have reduced the observed effect tend to increase the degree of certainty. For the evaluation, we used the GRADEproGDT tool available at https://gradepro.org/ (accessed on 5 August 2022).

### 4.4. Data Extraction

Once the publications of the systematic review and meta-analysis were selected, two reviewers (G-M. B.I. and P-C. J.) extracted the following data: last name of the first author, year of publication, study design, VR dose, duration of the intervention, sample size, age of the participants, glycemic parameters evaluated, and findings. For the meta-analysis, the mean (Ⴟ) and standard deviation (SD) of the pre- and post-intervention measurements of glucose, HbA1c, insulin, and HOMA-IR were extracted. The mean difference and SD were calculated using the following statistical formulas: 

Difference in means = meanpost-treatment − meanpre-treatment; 

For the calculation of the corresponding SD, the following formula was used:

$$\mathrm{SDdifference}=\sqrt{\left[\left({\mathrm{SDpreteatment}}^{2}+{\mathrm{SDposttreatment}}^{2}\right)-\left(2\times R\times \mathrm{SDpretreatment}\times \mathrm{SDposttreatment}\right)\right]}$$

where R = 0.8.

The Ⴟ, SD, mean differences, and their respective SD were calculated for the studies that did not report them, using suitable statistical methods [87,88]. The Ⴟ was calculated using the minimum and maximum values, the median, and the sample size (n), using the following formula:

Ⴟ = (minimum value + 2n + maximum value)/4

The SD was calculated with the following formula:

SD = √1/12[Ⴟ^2^ + (maximum value − minimum value)^2^]

In some cases, the SD was calculated from the standard error (SE) with the following formula:

SD = ES × √n.

Unit conversions were also performed to standardize the results reported in the studies. The glucose value in mmol/L was multiplied by 18 to convert to mg/dL. The conversion of insulin units from pmol/L to mIU/L was carried out by dividing pmol/L ÷ 6.945. The formula %HbA1c = (mmol/mol/10.929) + 2.15 was applied to convert HbA1c units to a percentage. To calculate the HOMA-IR index, the following formula was used:

HOMA-IR = [insulin (mIU/L) × glucose (mg/dL)]/405.

### 4.5. Statistic Analysis

For the meta-analyses, we used the inverse variance method to estimate the global effect of RV on each of the glycemic parameters (serum glucose, insulin, HbA1c, and HOMA-IR levels). This method gives greater weight to larger studies with smaller standard errors, while small studies with larger standard errors contribute less weight to the meta-analysis, thus minimizing imprecision in the estimatation of the overall effect. We also used the random effects model (Dersimonian and Laird method) to estimate the overall effect of RV supplementation on glycemic control, as this model accounts for intra- and inter-study heterogeneity. The heterogeneity of the included studies was assessed using the I^2^ statistic, which describes the percentage of variability present in the estimates of effect; we consider an I^2^ value < 40% as not considerable. To address heterogeneity, in addition to evaluating the influence of age and dose on the effects of RV on glycemic control, analyses were performed by dose (<250 mg/day, 250–500 mg/day, >500–1000 mg/day, and >1000 mg/day); by age (45–59 years and >60 years); and by dose and age (<250 mg/day, 45–59 years old; <250 mg/day, >60 years old; 250–500 mg/day, 45–59 years old; 250–500 mg/day, >60 years old; >500–1000 mg/day, 45–59 years; >500–1000 mg/day, >60 years; >1000 mg/day, 45–59 years; and >1000 mg/day, >60 years). In addition, sensitivity analyses were performed to assess the weight that each of the included studies contributed to the overall estimate. For this, all meta-analyses were carried out eliminating one study at a time. Egger’s test was performed to assess publication bias, which estimates the regression line between the precision of the studies and the standardized effect. For all analyses, *p* < 0.05 was considered statistically significant. The meta-analysis was performed with Review Manager version 5.4 software.

## 5. Conclusions

Our findings suggest that RV has a statistically significant dose–response effect on glucose concentrations, HbA1c percentage, and insulin levels in subjects with T2DM aged 45–59 years; however, HOMA-IR is not modified by the effect of oral administration of RV, except in subjects aged 60 years and over. In addition to this, there is not enough scientific evidence to propose a therapeutic dose, as individuals aged 60 years and over require higher doses than younger subjects to present the beneficial effects of RV; however, the small number of studies carried out in adults aged 60 years and over did not allow an adequate analysis of the influence of age on the effects of the polyphenolic compound in question. For this reason, is necessary to carry out more research in aged populations, as well as to elucidate the mechanisms through which the dose used and the age of the participants modify such effects.

## Figures and Tables

**Table 1:** Characteristics of clinical trials included in the review.

First Author (Year)	Study Design	Intervention	Population	Glycemic Parameters	Findings	Certainty
***Subjects 45–59 years old***						
Brasnyó et al. (2011) [23]	RCT double-blind	10 mg/day 4 weeks	19 men with T2DM 55 ± 9 years old	Insulin levels and HOMA-IR,	No changes in insulin levels, tendendency to decrease of HOMA-IR after RV administration	⨁◯◯◯ Very low
Bashmakov et al. (2014) [24]	RCT parallel-blind	100 mg/day 8 weeks	24 subjects with diabetic food 56 ± 9 years old	Glucose e insulin levels, HOMA-IR	Non-significant decrease of glucose in both study groups. No changes in insulin and HOMA-IR.	⨁◯◯◯ Very low
Imamura et al. (2017) [25]	RCT double-blind	100 mg/day 3 months	50 subjects with T2DM 57 ± 10 years old	Glucose levels and HbA1c	Non-significant changes after intervention	⨁◯◯◯ Very low
Mahjabeen et al. (2022) [26]	RCT double-blind	200 mg/day 24 weeks	110 subjects with T2DM 50 ± 11 years old	Glucose and insulin levels, HbA1c and HOMA-IR	Significant decrease in glucose and HbA1c (*p* < 0.05). Significant decrease in insulin and HOMA-IR (*p* = 0.001)	⨁⨁⨁◯ Moderate
Bhatt et al. (2012) [27]	RCT open-label	250 mg/day 3 months	57 subjects with T2DM 57 ± 9 years old	Glucose levels and HbA1c	Significant decrease in HbA1c (*p* < 0.05) after RV administration	⨁⨁◯◯ Low
Bhatt et al. (2013) [28]	RCT open-label	250 mg/day 6 months	57 subjects with T2DM 57 ± 9 years old	Glucose levels and HbA1c	Non-significant decrease in HbA1c and glucose levels	⨁⨁◯◯ Low
Javid et al. (2016) [29]	RCT double-blind	480 mg/day 4 weeks	43 subjects with T2DM and CP 50 ± 8 years old	Glucose and insulin levels, HOMA-IR	Significant decrease in insulin and HOMA-IR (*p* < 0.05). No significant changes in glucose levels	⨁⨁⨁◯ Moderate
Khodabandenlhoo et al. (2018) [30]	RCT double-blind	800 mg/day 8 weeks	45 subjects wit T2DM 57 ± 9 years old	Glucose and insulin levels, HbA1c, HOMA-IR	Significant decrease in glucose levels (*p* < 0.05). No significant changes in HbA1c, insulin levels, and HOMA-IR	⨁⨁◯◯ Low
Seyyedebrahimi et al. (2018) [31]	RCT double-blind	800 mg/day 8 weeks	46 subjects with T2DM 58 ± 6 years old	Glucose and insulin levels, HbA1c and HOMA-IR	Non-significant changes after RV administration	⨁⨁◯◯ Low
Abdollahi et al. (2019) [32]	RCT double-blind	1 g/day 8 weeks	71 subjects with T2DM and overweight 50 ± 7 years old	Glucose and insulin levels, HbA1c, HOMA-IR	Significant decrease in glucose (*p* = 0.03) and insulin levels (*p* = 0.02), improvement in HOMA-IR (*p* = 0.01). No significant changes in HbA1c	⨁⨁⨁◯ Moderate
Movahed et al. (2013) [33]	RCT double-blind	1 g/day 45 days	64 subjects with T2DM 52 ± 7 years old	Glucose and insulin levels, HbA1c and HOMA-IR	Significant decrease in glucose, insulin and HbA1c levels (*p* < 0.05). Improvement in HOMA-IR after RV administration	⨁⨁◯◯ Low
Goh et al. (2014) [34]	RCT double-blind	3 g/day 3 months	10 subjects with TD2M 56 ± 6 years old	Glucose and insulin levels, HbA1c, HOMA-IR	Tendency to decrease in HbA1c; no significant changes in HOMA-IR. No changes in glucose and insulin levels	⨁⨁⨁◯ Moderate
Sattarinezhad et al. (2019) [35]	RCT double-blind	500 mg/day 3 months	60 subjects with T2DM and albuminuria 57 ± 9 years old	Glucose and insulin levels, HbA1c and HOMA-IR	Significant decrease in glucose, insulin and HbA1c levels (*p* < 0.05). Improvement in HOMA-IR after RV administration	⨁⨁⨁◯ Moderate
***Subjects > 60 years old***						
Timmers et al. (2016) [36]	RCT double-blind cross-over	150 mg/day 4 weeks	16 subjects with T2DM 64 ± 4 years old	Glucose and insulin levels, HbA1c	Non-significant changes after RV administration	⨁⨁◯◯ Low
Bo et al. (2016) [37]	RCT double-blind	40, 500 mg/day 6 months	179 subjects with T2DM 65 ± 8 years old	Glucose and insulin levels, HOMA-IR, HbA1c	Non-significant changes between study groups	⨁⨁⨁◯ Moderate
Hoseini et al. (2019) [38]	RCT double-blind	500 mg/day 4 weeks	56 subjects with T2DM and CD 62 ± 9 years old	Glucose and insulin levels, HOMA-IR	Significant decrease in glucose and insulin levels (*p* = 0.01) and HOMA-IR (*p* = 0.001)	⨁⨁⨁◯ Moderate
Thazhath et al. (2016) [39]	RCT double-blind cross-over	1 g/day 5 weeks	14 subjects with T2DM 68 ± 2 years old	Glucose levels and HbA1c	Non-significant changes in glucose and HbA1c	⨁⨁◯◯ Low

Abbreviations: CD, coronary disease; CP, chronic periodontitis; HbA1c, glycated hemoglobin; HOMA-IR, insulin resistance; RCT, randomized clinical trial; RV, resveratrol; T2DM, type 2 diabetes mellitus.

**Table 2:** Analysis to evaluate the influence of age and dose on the effect of RV in T2DM.

Subgroup	No. of Trials	Effect Size	95% CI	*p*-Value	Heterogeneity (I^2^)	*p*-Value for I^2^
**Glucose −14.13 (−20.90,−7.36) *p* < 0.0001**						
**RV dosage (I^2^ = 58%; *p* = 0.07)**						
<250 mg/day	4	−5.41	−12.74, 1.93	0.15	42%	0.16
250–500 mg/day	5	−20.72	−30.62, −10.83	<0.0001	90%	<0.00001
>500–1000 mg/day	5	−16.40	−34.05, 1.25	0.07	91%	<0.00001
>1000 mg/day	1	3.60	−24.87, 32.07	0.80	----	----
**Age (I^2^ = 84%; *p* = 0.01)**						
45–59 years	12	−17.73	−25.93, −9.54	<0.0001	95%	<0.00001
≥60 years	3	−2.00	−11.29, 7.28	0.67	64%	0.06
**HbA1c −0.27 (−0.44, −0.10) *p* = 0.002**						
**RV dosage (I^2^ = 78%; *p* = 0.003)**						
<250 mg/day	4	−0.04	−0.27, 0.19	0.72	77%	0.005
250–500 mg/day	3	−0.58	−0.76, −0.39	<0.0001	98%	0.00001
>500–1000 mg/day	4	−0.26	−0.71, 0.19	0.25	81%	0.001
>1000 mg/day	1	−0.90	−2.00, 0.20	0.11	----	----
**Age (I^2^ = 95%; *p* = 0.0001)**						
45–59 years	10	−0.37	−0.54, −0.20	<0.0001	96%	0.0001
≥60 years	2	0.17	0.01, 0.34	0.04	0%	0.62
**Insulin −1.36 (−2.03, −0.68) *p* < 0.0001**						
**RV dosage (I^2^ = 0; %; *p* = 0.92)**						
<250 mg/day	4	−1.22	−1.73, −0.71	<0.0001	0%	0.56
250–500 mg/day	3	−1.55	−3.08, −0.03	0.05	81%	0.00
>500–1000 mg/day	3	−1.99	−5.19, 1.21	0.22	80%	0.006
>1000 mg/day	1	−0.40	−4.97, 4.17	0.86	----	----
**Age (I^2^ = 0%; *p* = 0.92)**						
45–59 years	9	−1.39	−2.21, −0.56	0.001	73%	0.0003
≥60 years	2	−1.32	−2.23, −0.41	0.005	69%	<0.0001
**HOMA-IR −0.77 (−1.60, 0.06) *p* = 0.07**						
**RV dosage (I^2^ = 26%; *p* = 0.26)**						
<250 mg/day	3	−0.41	−1.10, 0.29	0.25	63%	0.07
250–500 mg/day	3	−1.08	−2.16, 0.01	0.05	92%	0.00001
>500–1000 mg/day	4	−1.06	−2.95, 0.83	0.27	97%	0.00001
>1000 mg/day	1	0.31	−0.65, 1.26	0.53	----	----
**Age (I^2^ = 0%; *p* = 0.41)**						
45–59 years	9	−0.84	−1.83, 0.15	0.10	96%	0.00001
≥60 years	2	−0.40	−0.70, −0.11	0.007	0%	0.89

Abbreviations: CI, confidence interval; HbA1c, glycated hemoglobin; HOMA-IR, insulin resistance (homeostatic model); RV, resveratrol.

**Figure A1:**
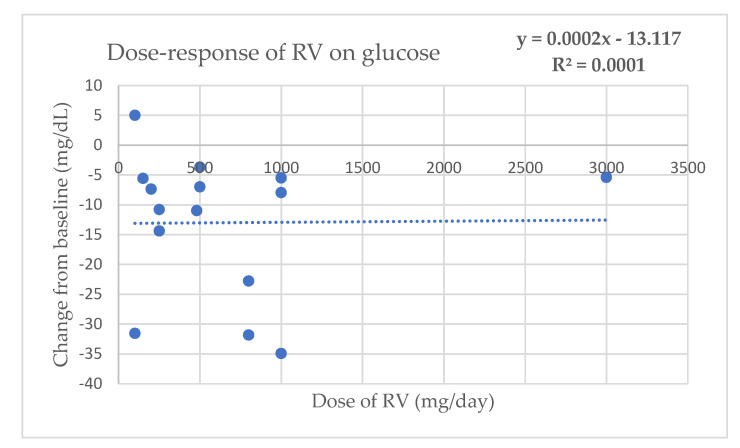
Dose–response regression analysis to assess the effect of RV on serum glucose levels.

**Figure A2:**
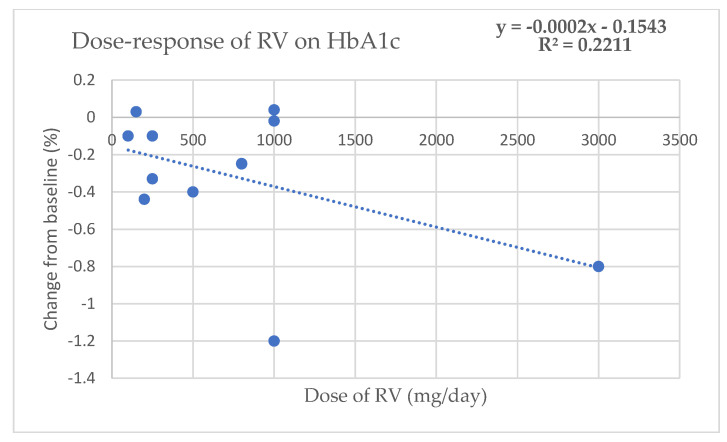
Dose–response regression analysis to assess the effect of RV on HbA1c.

**Figure A3:**
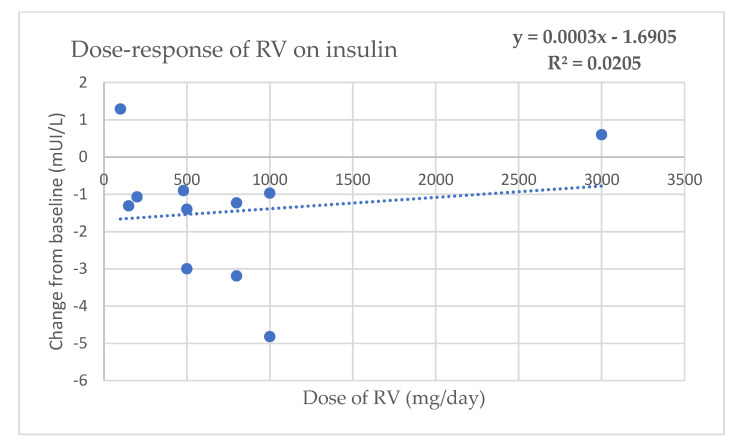
Dose–response regression analysis to assess the effect of RV on serum insulin levels.

**Figure A4:**
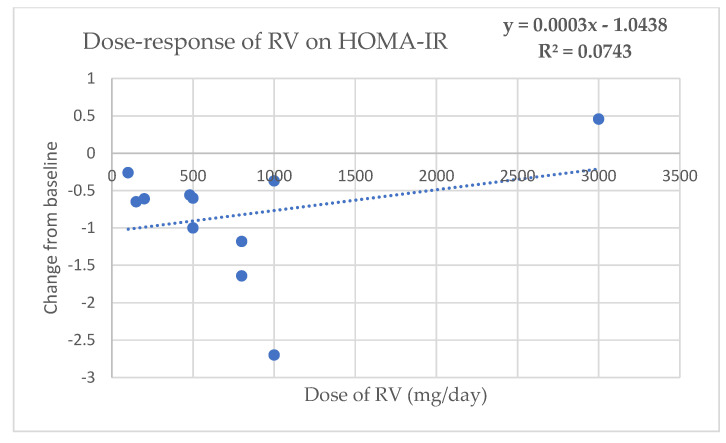
Dose–response regression analysis to assess the effect of RV on HOMA-IR.

## Data Availability

The data presented in this study are available on request from the corresponding author.

## Appendix A. Studies Retrieved from Databases that Were Excluded from the Systematic Review and Meta-Analysis

**Table A1:** Studies excluded from the systematic review.

Study	Reason for Exclusion
Abdollahi, et al. BMJ. 2019;9:e026337. Doi:10.1136/ bmjopen-2018-026337	It is a protocol
Arcanjo, et al. Biochim Biophys Acta Gen. 2018;1862:1938-1947. Doi: 10.1016/j.bbagen.2018.06.007	They do not evaluate glycemic parameters
Bo, et al. Acta Diabetol. 2018;55:331–3402018. Doi:10.1007/s00592-017-1097-4	They report only pre-treatment means of glycemic parameters
Bo, et al. Nutr Diabetes. 2018;8:51. Doi: 10.1038/s41387-018-0059-4	They do not evaluate glycemic parameters
Bo, et al. Acta Diabetol. 2017;54:499–507. Doi: 10.1007/s00592-017-0977-y	They do not report only pre- and post-treatment means of glycemic parameters
De Ligt, et al. Mol Metab. 2018;12: 39e47. Doi: 10.1016/j.molmet.2018.04.004	They do not evaluate glycemic parameters
Froghi, et al. IJEM. 2018;20:169–176.	Language other than English or Spanish
Javid, et al. Diabetes Metab Syndr. 2019;13:2769–2774. Doi: 10.1016/j.dsx.2019.07.042	They do not evaluate glycemic parameters
Pollack, et al. J Gerontol A Biol Sci Med Sci. 2017;72(12):1703–1709. Doi: 10.1093/gerona/glx041	They report only pre-treatment means of glycemic parameters
Saldanha, et al. J Ren Nutr. 2016. Doi: 10.1053/j.jrn.2016.06.005	They do not evaluate glycemic parameters
Tabatabaie, et al. Phytoter Res. 2020;34:2023–2031.Doi: 10.1002/ptr.6655	They report only pre-treatment means of glycemic parameters
Tomé-Carneiro et al. Pharmacol Res. 2013. Doi: 10.1016/j.phrs.2013.03.011	They use a grape extract
Toupchian, et al. Phytoter Res. 2021;35: 3205–3213. Doi: 10.1002/ptr.7031	They do not evaluate glycemic parameters
Wong, et al. Nutr Metabol Cardiovasc Dis. 2016. Doi: 10.1016/j.numecd.2016.03.003	They use a single dose of RV
Wong, et al. Nutrients. 2016, 8, 425. Doi:10.3390/nu8070425	They use a single dose of RV
Zhang, et al. Arterioscler Thromb Vasc Biol. 2017;37:A164. Doi: 10.1161/atvb.37.suppl_1.164	Only abstract available

## Appendix B. Documents Retrieved from Other Sources that Were Excluded from the Systematic Review and Meta-Analysis

**Table A2:** Records of clinical trials excluded from the systematic review.

Reason for Exclusion	Document Excluded
They do not meet the selection criteria (n = 19)	NCT04449198, NCT03762096, NCT03436992, NCT01997762, NCT03436992, IRCT201710108129N11, CTRI/2017/04/008384, NCT04449198, NCT00823381, NCT01593605, NCT02565979, NCT02129595, NCT02247596, NCT02834078, NCT01451918, NCT01714102, NCT03597568, NCT01150955, NCT03866005
They do not assess glycemic parameters (n = 8)	IRCT20171118037528N1, NCT01158417, NCT03762096, NCT01354977, NCT02502253, NCT02216552, NCT01302639, NCT02244879
Unpublished data (n = 7)	SLCTR/2018/019, IRCT201601022394N19, NCT01038089, NCT05172947IRCT201411112394N14, NCT02502253, NCT02549924,
Published data included in review (n = 11)	IRCT2016100716876N2, NCT02704494 (Sattarinezhad, 2019) [35], IRCT2015080223336N2 (Khodabandehloo, 2018) [30], IRCT2015072523336N1 (Seyyedebrahimi, 2018) [31], IRCT2015012420765N1 (Javid, 2016) [29], ACTRN12613000717752 (Thazhath, 2016) [39], IRCT201111198129N1 (Movahed, 2013) [33], CTRI/2011/05/001731 (Bhatt, 2012; Bhatt, 2013) [27,28], ACTRN12610000565044 (Basmakov, 2014) [24], NCT01677611 (Goh, 2014) [34], NCT01638780 (Timmers, 2016) [36].
Published data excluded from review (n = 3)	ACTRN12614000891628 (Wong, 2016), NCT01881347 (Zhang, 2017), NCT02244879 (Bo, 2018)
Language other than English or Spanish (n = 1)	IRCT20191203045588N1
Ongoing (n = 2)	CTRI/2022/05/042806, ISRCTN15172592
